# Comparative Genomics Insights Into the Evolutionary Disparities Between Nitroplast‐Evolved Ecotype UCYN‐A2 and Its Closest Relative UCYN‐A1

**DOI:** 10.1002/ece3.71739

**Published:** 2025-07-07

**Authors:** Shiyun Han, Sijia Zhang, Wen Ge, Jianke Yang, Hui Peng, Jinming Gao, Mengsa Zhang, Yingying Xiao, Dongsheng Du, Xianzhao Kan

**Affiliations:** ^1^ Anhui Provincial Key Laboratory of the Conservation and Exploitation of Biological Resources College of Life Sciences, Anhui Normal University Wuhu China; ^2^ Anhui Rural Revitalization Collaborative Technology Service Center Wuhu Institute of Technology Wuhu Anhui China; ^3^ School of Food and Bioengineering Wuhu Institute of Technology Wuhu Anhui China; ^4^ School of Basic Medical Sciences Wannan Medical College Wuhu Anhui China; ^5^ Department of Science and Technology, the First Affiliated Hospital of Wannan Medical College (Yijishan Hospital of Wannan Medical College) Wuhu Anhui China; ^6^ The Institute of Bioinformatics College of Life Sciences, Anhui Normal University Wuhu Anhui China

**Keywords:** bacterial evolution, codon aversion, codon usage bias, INDEL, molecular marker, nitroplast, UCYN‐A

## Abstract

UCYN‐A is a phenomenal diazotrophic cyanobacterium with significant ecological importance. UCYN‐A1 and UCYN‐A2 are the two most abundant ecotypes. Recently, the striking discovery of nitroplast, a novel N_2_‐fixing organelle in cultured *B. bigelowii*/UCYN‐A2 endosymbiont, indicated the possibility that UCYN‐A2 has evolved beyond endosymbiosis to an early phase of organellogenesis. This study addresses the following critical question: What evolutionary heterogeneity has emerged between UCYN‐A1 and UCYN‐A2? To investigate this issue, we comprehensively compared a total of seven genomes from UCYN‐A2 and UCYN‐A1. Under similar genome organizations, GC content, and gene composition, we still detected abundant genetic differences, including group–unique orthogroups, ANI below 85%, and 577 UCYN‐A2‐unique INDELs in single‐copy orthologous genes (SCOGs). Moreover, we also focused on the orthologous genes of 40 metabolic‐pathway genes in nitroplast. In addition to high‐informative SNPs and INDELs possessing distinct interlineage differences, we traced abundant codon usage “signatures” that serve as lineage‐unique molecular markers. Most notably, we successfully established a strain‐level identification map for UCYN‐A strains using codon aversion motifs, which represents the first case study of this approach in bacteria. In summary, all the comparative results reported here collectively indicate that UCYN‐A1 and UCYN‐A2 have evolved remarkable genomic heterogeneities. Furthermore, the findings of this work will definitely promote our current understanding of codon aversion and the evolution of UCYN‐A.

## Graphical abstract

This paper conducted comparative genomic analyses among seven UCYN‐A strains in genome content, gene structure, and key metabolic protein genes. In general, our results demonstrated considerable disparities between UCYN‐A1 and UCYN‐A2, providing insights into the evolution of UCYN‐A and nitroplast.

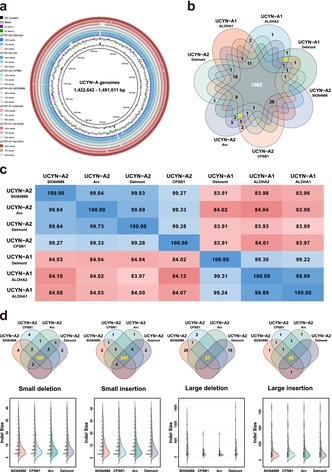

## 1 Introduction

Biological nitrogen fixation (BNF) plays an indispensable part in maintaining life on Earth (Bombar et al. 2014). It has been widely accepted that BNF limits primary production more than phosphorus (Falkowski 1997; Vitousek and Howarth 1991). To the best of our knowledge, all documented diazotrophs were restricted to bacteria and a few archaea (Postgate 1998). In fact, this observation is consistent with the early evolution of BNF in prokaryotes, which was presumably driven by the imbalance between the decreased nitrogen and the increased microbial biomass (Raymond et al. 2004; Towe 2002). Noteworthily, the BNF has been assigned into two major types: symbiotic (Granhall 1981) and free‐living BNF (Reed et al. 2011). In terrestrial natural ecosystems, symbiotic BNF consistently accounts for a greater proportion of nitrogen fixation than free‐living BNF (Davies‐Barnard and Friedlingstein 2020). Diazotrophs usually enhance fixation efficiency by establishing symbiotic relationships with land plants (Mylona et al. 1995), with classic examples including rhizobia‐legumes (Hellriegel and Wilfarth 1888), *Nostoc*‐*Gunnera* (Silvester 1976), and *Frankia*‐actinorhizal symbioses (Torrey and Tjepkema 1979). In contrast, nitrogen fixation in oceanic areas appears to be dominated by the free‐living BNF, especially the most widespread *Trichodesmium* (Zehr 1995). Meanwhile, symbiotic BNF has also been discovered between diazotrophs and algae, such as the symbiont of *Richelia*/*Calothrix* with diatoms (Villareal 1992; Janson et al. 1999) and *Candidatus* Atelocyanobacterium thalassa (UCYN‐A) with haptophytes (Thompson et al. 2012).

UCYN‐A is well known for the first case of establishing a mutualistic symbiosis with prymnesiophyte algae (*Braarudosphaera bigelowii*) (Thompson et al. 2012; Hagino et al. 2013; Suzuki et al. 2021). It should be noted that this diazotrophic cyanobacterium is phenomenal in several aspects. Differing from other diazotrophs, UCYN‐A has undergone a relatively large genome reduction, evidenced by the widespread loss of genes involved in metabolic pathways (Tripp et al. 2010). Moreover, another characteristic is its remarkably global distribution (Díez et al. 2012; Rees et al. 2009; Langlois et al. 2008; Needoba et al. 2007; Farnelid et al. 2016), in contrast to the mainly tropical/subtropical distribution of other diazotrophs (e.g., the dominant *Trichodesmium*) (Letelier and Karl 1996; Sohm et al. 2011). Although formerly considered to have low genetic diversity within the lineage (Tripp et al. 2010), after years of effort, UCYN‐A is widely accepted to harbor at least six distinct ecotypes (UCYN‐A1 to UCYN‐A6) (Turk‐Kubo et al. 2017; Farnelid et al. 2016; Thompson et al. 2014), with the possibility of even more (Henke et al. 2018). Notably, UCYN‐A1 and UCYN‐A2 are the most abundant sublineages among them (Turk‐Kubo et al. 2017). Moreover, they are the only two ecotypes with explicitly described hosts, while those of other UCYN‐As are still unknown (Cornejo‐Castillo et al. 2019).

As far as we know, a series of variations have been revealed between UCYN‐A1 and UCYN‐A2 (Table 1), including host specificity (Thompson et al. 2012; Thompson et al. 2014; Cornejo‐Castillo et al. 2019), the scale of symbionts held by the host (Bombar et al. 2014; Thompson et al. 2014; Cornejo‐Castillo et al. 2019; Suzuki et al. 2021), symbiont–host association patterns (Suzuki et al. 2021; Fletcher‐Hoppe et al. 2023), symbiosis nitrogen fixation rates (Turk‐Kubo et al. 2021), and genomic content (Bombar et al. 2014). Furthermore, from a remarkable recent discovery in cultured *B. bigelowii*/UCYN‐A2 endosymbiont, this UCYN‐A2 was identified as a novel N_2_‐fixing organelle called the “nitroplast,” opening new avenues for research in this field. Coale et al. (2024b) rigorously investigated the organelle qualifications of UCYN‐A2 by examining its architectural integration into host cell, synchronized replication and fission with host organelles, as well as the reception of host‐encoded proteins. It is also noteworthy that the authors depicted the metabolic‐pathway genetic map of the nitroplast, involving genes from both UCYN‐A and *B. bigelowii*. Based on these points, UCYN‐A2 has evolved beyond endosymbiosis to an early phase of organellogenesis (Macorano and Nowack 2021; Coale et al. 2024b). There might be a reasonably emerging mystery here: what evolutionary heterogeneity has emerged between UCYN‐A1 and UCYN‐A2?

**TABLE 1:** Summary of the variations between UCYN‐A1 and UCYN‐A2.

Aspects with divergence	UCYN‐A1	UCYN‐A2
Host specificity	Diameter at 1–3 μm (Thompson et al. 2014; Cornejo‐Castillo et al. 2019)	Diameter at 4–10 μm (Cabello et al. 2016)
Housed‐symbiont scales of hosts	1–2 per host cell (Thompson et al. 2014; Cornejo‐Castillo et al. 2019)	1 per host cell (microscopy result) (Suzuki et al. 2021) 4–10 per host cell (DNA sequencing result) (Bombar et al. 2014; Cornejo‐Castillo et al. 2019)
Symbiont–host association patterns	Strong (Fletcher‐Hoppe et al. 2023)	Weak (Host can dissociate symbiont) (Suzuki et al. 2021)
Gene content	Lacking genes for cell shape and wall (Bombar et al. 2014)	Possessing genes for cell shape and wall (Bombar et al. 2014)
Symbiosis nitrogen fixation rates	6.6 ± 8.8 fmol N cell^−1^ day^−1^ (Turk‐Kubo et al. 2021)	151.1 ± 112.7 fmol N cell^−1^ day^−1^ (Turk‐Kubo et al. 2021)

Codon usage bias (CUB), the uneven usage of synonymous codons, has long been considered one important tool for gaining evolutionary insights into organisms (Salim and Cavalcanti 2008; Ding, Bi, et al. 2022; Iriarte et al. 2021; Leffler et al. 2012; Parvathy et al. 2022). To date, a set of indices has been widely employed for CUB assessment, such as relative synonymous codon usage (RSCU) (Sharp and Li 1986), effective number of codons (ENC) (Wright 1990), and the parity rule 2 (PR2) plot (Sueoka 1995). Importantly, the high efficiency of these indices in recognizing interlineage disparities has been iteratively examined in various organisms. For instance, this has been observed in Crenarchaea and Euryarchaea (Archaea) (Baruah et al. 2016), cyanobacteria (Bacteria) (Prabha et al. 2017), ciliated protozoa (Protista) (Fu et al. 2023), *Aspergillus* (Fungi) (Hugaboom et al. 2023), Saxifragaceae (Plantae) (Bi et al. 2023), and Certhioidea (Animalia) (Ding, Bi, et al. 2022). Apart from preferred codons, the codons that are avoided have recently attracted attention. The codon aversion motif (CAM) was first proposed as a novel phylogenetic character system by Miller et al. (Miller et al. 2017). Subsequently, our research group made great efforts to examine its potential as a unique marker in both plant (Ding, Han, et al. 2022; Han, Bi, et al. 2022; Han, Wang, et al. 2022; Han, Zhang, et al. 2024; Yang et al. 2023; Zhang, Han, et al. 2024) and animal genes (Ding, Bi, et al. 2022; Han, Ding, et al. 2024), although bacterial genes have not yet been involved. To date, a detailed illustration or comprehensive analysis of the CUB and CAM patterns in UCYN‐A has not been reported. In‐depth analyses are vitally needed to better understand both UCYN‐A evolution and bacterial CAM.

To advance the understanding of this issue, our study aimed to provide genetic evidence from all seven published UCYN‐A1/UCYN‐A2 genomes. Here, we reannotated the three A1 and four A2 genome sequences and extracted all the SCOGs. Through comprehensive analyses, this work endeavors to elucidate the disparities between A1 and A2 ecotypes in several aspects, including (1) overall gene content, (2) nucleotide compositions of all SCOGs, and (3) sequence variations, codon usage, and aversion patterns of the key metabolic‐pathway genes in nitroplast. Taken together, we hope our results will offer valuable insights into the evolutionary relationships among the UCYN‐A lineages.

## 2 Materials and Methods

### 2.1 Genome Sampling, Reannotation, Overall Genomic and Genetic Comparisons

The current study collected all seven publicly available UCYN‐A genomes, comprising three A1 and four A2 ecotypes (Table S1). We first performed an average nucleotide identity (ANI) analysis using JSpeciesWS, employing the BLASTn algorithm (ANIb) (Richter et al. 2016) as the basis for calculation, while avoiding the MUMMER algorithm due to its reported limitation (Li et al. 2015). Next, the BRIG package contributed to the comparative genome‐map depiction (Alikhan et al. 2011). Subsequently, all seven retrieved genomes were reannotated using Prokka (Seemann 2014). To facilitate data extraction, we then developed a Python script for simultaneous retrieval of target sequences, including proteins, protein‐coding genes (PCGs), transfer RNA (tRNA) genes, and ribosomal RNA (rRNA) genes. The GC content was assessed using MEGAX (Kumar et al. 2018). Furthermore, we created a Venn diagram in R to visualize the unique and shared genes among the UCYN‐A1/A2 (R Core Team 2013).

### 2.2 Extraction and Sequence Analyses of the Single‐Copy Orthologous Genes

To identify the SCOGs among the seven UCYN‐A genomes, we employed the OrthoFinder program (Emms and Kelly 2019). All target sequences were extracted and deposited in Figshare (https://doi.org/10.6084/m9.figshare.28200554.v1). For nucleotide alignment, we used MAFFT v7.505 (Katoh and Standley 2013), followed by manual adjustments in BioEdit v7.2.5 (Hall 1999) to ensure compliance with previously established alignment rules (Simmons and Ochoterena 2000; Lohne and Borsch 2005; Borsch et al. 2003). Subsequently, a self‐written Python script was used to detect insertion–deletion polymorphisms (INDELs) among the SCOGs of UCYN‐A1/A2 (deposited in https://github.com/Hesseatti/Python‐Script/MutIndelScan.py). The resulting data were tabulated with information on the located strain, positions, and lengths of the identified INDELs, as well as the specific sequences. Furthermore, based on bar, box, and violin plots, the INDELs' distributions and sizes were graphically displayed using R (R Core Team 2013). According to the method proposed by Wetterbom et al. (Wetterbom et al. 2006), for each SCOGs, the constituent INDEL divergences were estimated by the ratio of indel sizes to the total alignment size.

### 2.3 Comparative Analyses Among the Metabolic‐Pathway Genes

#### 2.3.1 Sequence Acquisition of the Metabolic‐Pathway Genes From the Seven UCYN‐A Strains

Coale et al. (2024b) summarized a set of UCYN‐A‐encoded genes involved in key metabolic pathways, which were critical and informative targets for examining the potential conservation and divergence between the ecotypes. Firstly, by combining the data S7 and proteomics data from Coale et al. (2024b), Coale et al. (2024a), we obtained a total of 40 well‐annotated metabolic‐pathway protein sequences after carefully verifying the sequence integrity. Next, OrthoFinder was used to identify SCOGs of these proteins using a dataset consisting of the whole protein sequences of the seven UCYN‐A1/A2 strains. We then extracted the corresponding coding DNA sequences (CDSs). After manually checking the obtained CDSs, a matrix of the 40 metabolic‐pathway genes was finally established (deposited in Figshare in https://doi.org/10.6084/m9.figshare.28200599.v1).

#### 2.3.2 Single Nucleotide Polymorphism (SNP) and INDEL Analyses of the Obtained Metabolic‐Pathway Genes

The SNP and INDELs in the established gene matrix were explored using the previously mentioned custom‐written script that is mentioned above. Note that 40 metabolic‐pathway genes could be assigned to nine different pathways. Further analyses of SNP data were performed in two aspects: a general comparison of SNP frequencies across different pathways and a detailed examination of specific SNP compositions for all genes. For INDEL analysis, we extracted gene segments containing the identified INDEL regions and visualized them using SequenceTubeMap (Beyer et al. 2019).

#### 2.3.3 Codon Usage Bias and Codon Aversion Motif Analyses in Metabolic‐Pathway Genes

According to standard protocols for CUB and CAM analyses, we selected over‐300‐bp CDSs, with terminal codons removed (Karlin et al. 1998; Yang et al. 2014; He et al. 2016; Han, Wang, et al. 2022; Han, Bi, et al. 2022; Ding, Bi, et al. 2022; Bi et al. 2023).

Our codon usage analysis focused on three commonly used indicators: (1) Relative Synonymous Codon Usage (RSCU) (value of each tested codon measures the ratio of factual to expected frequency), (2) effective number of codons (ENC) (value of each tested gene indicates the internal degree of codon preference), and (3) parity rule 2 (PR2) plot (point's position implies the impacts from mutation or selection on those 3rd positions in codons). As for detailed methodologies, the calculations of RSCU and ENC were realized using CodonW v1.4.4 (Peden 2000). Meanwhile, the abscissa and ordinate axes of the PR2 plot denote GC and AU biases, calculated by G3/(G3 + C3) and A3/(A3 + U3), respectively (Galtier and Lobry 1997; Sueoka 1999). Therein, we wrote a simple Python script to count the nucleotide content at the 3rd positions in codons (deposited in https://github.com/Hesseatti/Python‐Script/PR2Scan.py). Afterwards, all the corresponding graphic presentations were performed in ggplot2 (Wickham 2011).

Codon aversion basically denotes the codons that are not in use (Miller et al. 2017; Miller et al. 2020). Therefore, CAMs can be theoretically traced by the condition of RSCU = 0. We accordingly identified all the aversive codons in the metabolic‐pathway genes of UCYN‐A1/A2. The comparative results among all seven UCYN‐A strains were presented in an organizational chart.

## 3 Results

### 3.1 Similarities and Disparities of General Features, and Average Nucleotide Identities Among the Seven UCYN‐A Genomes

**FIGURE 1:**
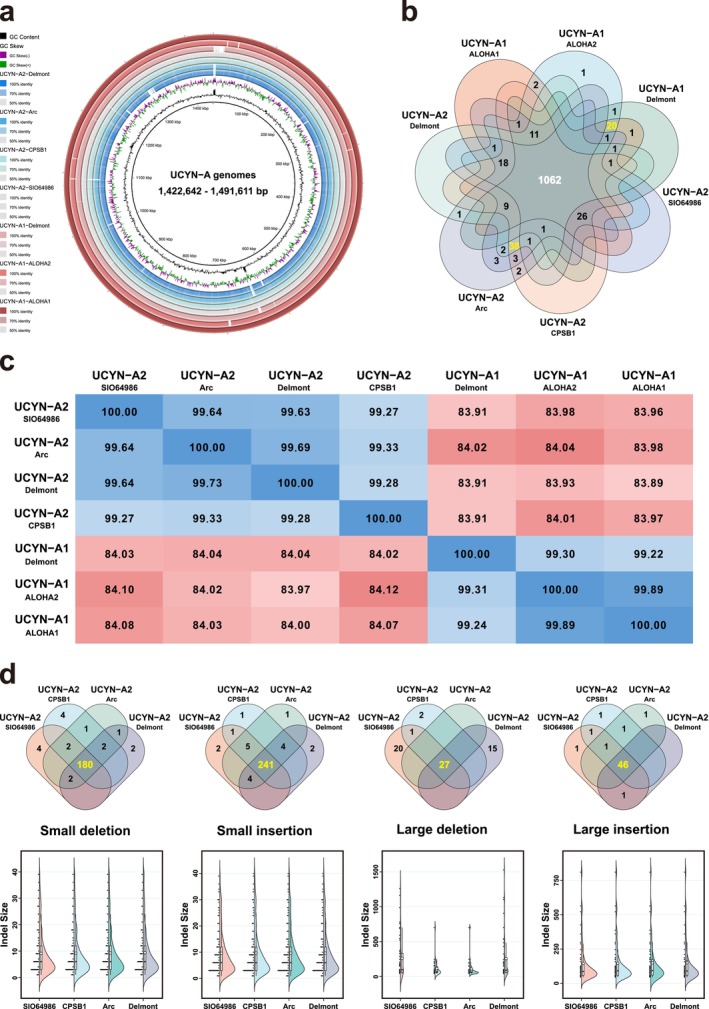
(a) Circular representation of the BLAST results among the sequences. From the outermost to the innermost, the concentric circles represented the genome sequences of UCYN‐A1‐ALOHA1, UCYN‐A1‐ALOHA2, UCYN‐A‐Delmont, UCYN‐A2‐SIO64986, UCYN‐A2‐CPSB1, UCYN‐A2‐Arc, and UCYN‐A2‐Delmont, respectively. (b) Venn map of the identified 1207 PCG orthogroups among all seven UCYN‐A strains. The numbers of unique PCGs for UCYN‐A1/A2 were marked with yellow. (c) Pairwise ANIb calculations among the seven strains. (d) Statistics of the 577 INDELs across the four UCYN‐A2 strains. The Venn plots showed the numbers of shared and unique INDELs in various types. The bar plot on the left shows the number of INDELs across different length categories. The central boxplot summarizes the distribution of INDEL lengths, where the box represents the interquartile range (IQR), the line inside the box denotes the median, whiskers extend to 1.5 times the IQR, and individual dots represent outliers. The violin plot on the right combines a kernel density estimation of the distribution with an embedded boxplot, providing both the data spread and distribution shape. These visualizations together provide a comprehensive view of INDEL length characteristics.

In general, a high degree of similarity was discovered in the genomic organizations among the seven UCYN‐A genomes. As depicted in Figure 1a, a circular representation of the BLAST results among the sequences revealed similar synteny patterns. With a size range of 1,422,642 – 1,491,611 bp, the UCYN‐A1 and UCYN‐A2 genomes shared overall strong similarities in GC content, gene number, and gene composition (Table 2). Such conservation was also supported by the results of Bombar et al. (2014). As for the GC content among the seven strains, the standard deviation (SD) values of the protein‐coding, rRNA, and tRNA genes were 0.0005164, 0.0004880, and 0.001345, respectively. The number of rRNA genes was consistently six across the seven genomes, while those of tRNA genes fell between 35 and 37. For PCGs, the gene counts of the seven strains ranged from 1220 to 1291 (Table 2).

**TABLE 2:** General genome features of the seven UCYN‐A genomes.

Statistics	UCYN‐A1‐ALOHA1	UCYN‐A1‐ALOHA2	UCYN‐A1‐Delmont	UCYN‐A2‐SIO64986	UCYN‐A2‐CPSB1	UCYN‐A2‐Arc	UCYN‐A2‐Delmont
Genome size	1,443,806	1,489,669	1,422,642	1,485,499	1,491,611	1,480,855	1,459,650
Number of contigs	1 (complete)	47	44	52	1 (complete)	5	46
Total genes	1237	1291	1223	1251	1251	1252	1220
Number of protein‐coding genes	1194	1248	1181	1208	1208	1211	1179
GC% of protein‐coding genes	33.1%	33.0%	33.0%	33.1%	33.1%	33.1%	33.1%
Number of rRNA genes	6	6	6	6	6	6	6
GC% of rRNA genes	52.6%	52.6%	52.6%	52.6%	52.5%	52.5%	52.6%
Number of tRNA genes	37	37	36	37	37	35	35
GC% of tRNA genes	56.6%	56.3%	56.7%	56.7%	56.6%	56.6%	56.6%

Significantly, we totally identified 1207 PCG orthogroups among all seven UCYN‐A strains (Table S2 and Figure 1b). Therein, 1062 (87.99%) were shared by all strains, 20 (1.66%) were exclusive to the three UCYN‐A1 strains, and 38 (3.15%) were unique to the UCYN‐A2 group. Of these 58 unique orthogroups, 42 (72.41%) encoded proteins with unknown functions, whereas the remaining 16 (27.59%) had certain annotated functions (Table 3). Note that compared with the work of Bombar et al. (2014) (detecting 1159 orthologous genes), inclusion of more samples decreased the number of orthologous genes between UCYN‐A1 and UCYN‐A2.

**TABLE 3:** Annotation of the 58 unique genes of UCYN‐A1 and UCYN‐A2 groups.

Type	Orthogroup ID	Gene ID	Functional annotation
Unique genes of UCYN‐A1 group (20)	OG0001171	OENGLBIA_00023, BGOCFPNC_00521, JIJLMCDE_00309	Hypothetical protein, function unknown
	OG0001172	OENGLBIA_00025, BGOCFPNC_00523, JIJLMCDE_00311	Restriction endonuclease
	OG0001173	OENGLBIA_00443, BGOCFPNC_00847, JIJLMCDE_00969	HAS barrel domain protein
	OG0001174	OENGLBIA_00466, BGOCFPNC_00261, JIJLMCDE_01039	Hypothetical protein, function unknown
	OG0001175	OENGLBIA_00508, BGOCFPNC_00133, JIJLMCDE_01204	Hypothetical protein, function unknown
	OG0001176	OENGLBIA_00544, BGOCFPNC_00555, JIJLMCDE_00552	Hypothetical protein, function unknown
	OG0001177	OENGLBIA_00552, BGOCFPNC_00170, JIJLMCDE_00268	NurA domain‐containing protein
	OG0001178	OENGLBIA_00728, BGOCFPNC_01270, JIJLMCDE_00019	Hypothetical protein, function unknown
	OG0001179	OENGLBIA_00765, BGOCFPNC_00330, JIJLMCDE_00056	Peroxiredoxin
	OG0001180	OENGLBIA_00832, BGOCFPNC_00936, JIJLMCDE_01185	Hypothetical protein, function unknown
	OG0001181	OENGLBIA_00878, BGOCFPNC_00245, JIJLMCDE_00318	Hypothetical protein, function unknown
	OG0001182	OENGLBIA_00905, BGOCFPNC_00218, JIJLMCDE_00840	Transcriptional regulator, GntR family
	OG0001183	OENGLBIA_00960, BGOCFPNC_00713, JIJLMCDE_01000	Hypothetical protein, function unknown
	OG0001184	OENGLBIA_01054, BGOCFPNC_00619, JIJLMCDE_00240	NAD‐dependent aldehyde dehydrogenase
	OG0001185	OENGLBIA_01099, BGOCFPNC_00113, JIJLMCDE_00194	Hypothetical protein, function unknown
	OG0001186	OENGLBIA_01146, BGOCFPNC_00067, JIJLMCDE_00148	Hypothetical protein, function unknown
	OG0001187	OENGLBIA_01154, BGOCFPNC_00058, JIJLMCDE_00140	Hypothetical protein, function unknown
	OG0001188	OENGLBIA_01168, BGOCFPNC_00044, JIJLMCDE_00126	Hypothetical protein, function unknown
	OG0001189	OENGLBIA_01173, BGOCFPNC_00039, JIJLMCDE_00121	Putative ATPase
	OG0001190	OENGLBIA_01174, BGOCFPNC_00038, JIJLMCDE_00119	Hypothetical protein, function unknown
Unique genes of UCYN‐A2 group (38)	OG0001133	LOLNPPPD_00002, ICNFDDEA_00626, LNCGHJMK_00733, GANCLMNP_00051	Hypothetical protein, function unknown
	OG0001134	LOLNPPPD_00054, ICNFDDEA_00677, LNCGHJMK_00785, GANCLMNP_00786	Hypothetical protein, function unknown
	OG0001135	LOLNPPPD_00063, ICNFDDEA_00686, LNCGHJMK_00794, GANCLMNP_00777	Hypothetical protein, function unknown
	OG0001136	LOLNPPPD_00065, ICNFDDEA_00688, LNCGHJMK_00796, GANCLMNP_00775	Hypothetical protein, function unknown

	OG0001137	LOLNPPPD_00134, ICNFDDEA_00757, LNCGHJMK_00865, GANCLMNP_00471	Hypothetical protein, function unknown
	OG0001138	LOLNPPPD_00195, ICNFDDEA_00816, LNCGHJMK_00924, GANCLMNP_00530	Glucosylglycerol‐phosphate phosphatase
	OG0001139	LOLNPPPD_00207, ICNFDDEA_01243, LNCGHJMK_00376, GANCLMNP_01125	Hypothetical protein, function unknown
	OG0001140	LOLNPPPD_00282, ICNFDDEA_01167, LNCGHJMK_00301, GANCLMNP_00213	Hypothetical protein, function unknown
	OG0001141	LOLNPPPD_00396, ICNFDDEA_00284, LNCGHJMK_00415, GANCLMNP_00756	Hypothetical protein, function unknown
	OG0001142	LOLNPPPD_00410, ICNFDDEA_00270, LNCGHJMK_00429, GANCLMNP_00170	Hypothetical protein, function unknown
	OG0001143	LOLNPPPD_00432, ICNFDDEA_00247, LNCGHJMK_00451, GANCLMNP_00148	Hypothetical protein, function unknown
	OG0001144	LOLNPPPD_00458, ICNFDDEA_00167, LNCGHJMK_00531, GANCLMNP_00245	Beta‐lactamase hydrolase‐like protein
	OG0001145	LOLNPPPD_00465, ICNFDDEA_00160, LNCGHJMK_00538, GANCLMNP_00252	Hypothetical protein, function unknown
	OG0001146	LOLNPPPD_00528, ICNFDDEA_00098, LNCGHJMK_00601, GANCLMNP_00681	Aminomethyltransferase, AMT
	OG0001147	LOLNPPPD_00575, ICNFDDEA_00051, LNCGHJMK_00648, GANCLMNP_00634	Hypothetical protein, function unknown
	OG0001148	LOLNPPPD_00598, ICNFDDEA_00414, LNCGHJMK_01062, GANCLMNP_00563	Hypothetical protein, function unknown
	OG0001149	LOLNPPPD_00633, ICNFDDEA_00449, LNCGHJMK_01027, GANCLMNP_00384	Hypothetical protein, function unknown
	OG0001150	LOLNPPPD_00649, ICNFDDEA_01060, LNCGHJMK_00194, GANCLMNP_01006	Hypothetical protein, function unknown
	OG0001151	LOLNPPPD_00688, ICNFDDEA_01020, LNCGHJMK_00154, GANCLMNP_00828	Hypothetical protein, function unknown
OG0001152	LOLNPPPD_00689, ICNFDDEA_01019, LNCGHJMK_00153, GANCLMNP_00829	Hypothetical protein, function unknown

	OG0001153	LOLNPPPD_00690, ICNFDDEA_01018, LNCGHJMK_00152, GANCLMNP_00830	Hypothetical protein, function unknown
	OG0001154	LOLNPPPD_00766, ICNFDDEA_00047, LNCGHJMK_00652, GANCLMNP_00630	Hypothetical protein, function unknown
	OG0001155	LOLNPPPD_00893, ICNFDDEA_00932, LNCGHJMK_00063, GANCLMNP_00321	Rod shape‐determining protein MreB
	OG0001156	LOLNPPPD_00894, ICNFDDEA_00931, LNCGHJMK_00062, GANCLMNP_00322	Hypothetical protein, function unknown
	OG0001157	LOLNPPPD_00895, ICNFDDEA_00930, LNCGHJMK_00061, GANCLMNP_00323	Hypothetical protein, function unknown
	OG0001158	LOLNPPPD_00923, ICNFDDEA_00609, LNCGHJMK_00716, GANCLMNP_00034	Hypothetical protein, function unknown
	OG0001159	LOLNPPPD_00971, ICNFDDEA_00827, LNCGHJMK_00935, GANCLMNP_00541	Hypothetical protein, function unknown
	OG0001160	LOLNPPPD_01042, ICNFDDEA_01095, LNCGHJMK_00229, GANCLMNP_00901	Hypothetical protein, function unknown
	OG0001161	LOLNPPPD_01084, ICNFDDEA_00497, LNCGHJMK_00979, GANCLMNP_01186	Hypothetical protein, function unknown
	OG0001162	LOLNPPPD_01112, ICNFDDEA_00985, LNCGHJMK_00116, GANCLMNP_00739	Folate‐biopterin transporter
	OG0001163	LOLNPPPD_01127, ICNFDDEA_00871, LNCGHJMK_00002, GANCLMNP_00260	RNA polymerase sigma factor RpoD
	OG0001164	LOLNPPPD_01128, ICNFDDEA_00872, LNCGHJMK_00003, GANCLMNP_00261	RNA polymerase sigma factor SigA2
	OG0001165	LOLNPPPD_01151, ICNFDDEA_00895, LNCGHJMK_00026, GANCLMNP_00869	Hypothetical protein, function unknown
	OG0001166	LOLNPPPD_01158, ICNFDDEA_00356, LNCGHJMK_01119, GANCLMNP_01084	Hypothetical protein, function unknown
	OG0001167	LOLNPPPD_01219, ICNFDDEA_00392, LNCGHJMK_01084, GANCLMNP_00931	Hypothetical protein, function unknown
	OG0001168	LOLNPPPD_01234, ICNFDDEA_01118, LNCGHJMK_00252, GANCLMNP_00346	Tetratricopeptide repeat protein
	OG0001169	LOLNPPPD_01238, ICNFDDEA_01115, LNCGHJMK_00249, GANCLMNP_00343	Alkyl hydroperoxide reductase C
OG0001170	LOLNPPPD_01251, ICNFDDEA_00908, LNCGHJMK_00039, GANCLMNP_00856	Hypothetical protein, function unknown

To assess the overall genome relatedness, we calculated the pairwise ANIb values among the seven UCYN‐A strains. It was worth noting that Figure 1c obviously demonstrated two types of results: (1) all ANIb values of UCYN‐A1 versus UCYN‐A2 fell below 85% (83.89% – 84.12%), and (2) the ANIb values among strains within the same ecotype (either UCYN‐A1 or UCYN‐A2) all exceeded 99% (99.22% – 99.69%).

### 3.2 Abundant INDEL Events in the Single‐Copy Orthologous Genes Between the UCYN‐A1 and UCYN‐A2 Groups

Among the seven UCYN‐A genomes, a total of 1020 single‐copy orthologous genes were identified and used for further analyses (Table S3). With the consensus sequences of the UCYN‐A1 strains generated in BioEdit v7.2.5 (Hall 1999) as references, the aligned SCOGs matrices strikingly provided 577 INDELs across the four UCYN‐A2 strains (Table S4 and Figure 1d). Following the empirical classification of INDELs proposed by Zhao and Zhao (2015), we classified these events into four types: small (≤ 40 bp) and large (> 40 bp) insertions and deletions. Among the 577 INDELs, small insertions (261) occupied the largest proportion at 45.23%, followed by small deletions (198, 34.32%), large deletions (65, 11.27%), and large insertions (53, 9.19%) (Figure 1d).

As obviously shown in Table 4, the UCYN‐A2 strains shared high identity throughout the four classes of INDELs (86.80%–92.34%), with the exception of the large deletion category (41.54%). The size distributions of the INDELs were also in accordance with this pattern, with high similarities in small deletion, small insertion, and large insertion types (Figure 1d). Furthermore, patterns of INDEL divergence were rather similar between the small deletion and insertion categories. In contrast, great divergence was observed between the large INDEL groups. Significantly, one interesting finding is that, for both small and large categories, the average INDEL divergence of insertions was higher than that of deletions.

**TABLE 4:** INDEL identity and INDEL divergence calculation across the 1020 SCOG alignments among the four UCYN‐A2 strains, with the consensus sequences of the three UCYN‐A1 strains as references.

INDEL categories	INDEL identity (shared INDEL number/total INDEL number)	INDEL divergence (indel size/total alignment size) (mean ± SD)
Small deletion	90.91%	0.0109 ± 0.0128
Small insertion	92.34%	0.0133 ± 0.0236
Large deletion	41.54%	0.1496 ± 0.1174
Large insertion	86.80%	0.1902 ± 0.1791

### 3.3 Considerable Disparities Among the Metabolic‐Pathway Genes Between the UCYN‐A1 and UCYN‐A2 Groups

Most recently, Coale et al. (2024b) clearly revealed a series of UCYN‐A‐coding genes involved in nitroplast metabolic pathways. Using 40 well‐annotated protein sequences from nine pathways as references, we identified the corresponding orthologous genes in our investigated seven UCYN‐A strains (Table 5). Here we present a comprehensive comparison results between the UCYN‐A1 and UCYN‐A2 groups.

**TABLE 5:** Size and GC content of the orthologous genes for the nitroplast metabolic pathway genes traced in seven UCYN‐A strains.

Pathway genes	UCYN‐A1‐ALOHA1	UCYN‐A1‐ALOHA2	UCYN‐A1‐Delmont	UCYN‐A2‐SIO64986	UCYN‐A2‐CPSB1	UCYN‐A2‐Arc	UCYN‐A2‐Delmont	Mean value of UCYN‐A1	Mean value of UCYN‐A2
C5 isoprenoid biosynthesis	Size (bp)/GC content (%)								
Dsx	1911/38.62	1911/38.62	1911/38.30	1911/38.10	1911/38.04	1911/38.04	1911/38.04	1911/38.51	1911/38.06
Dxr	213/24.88	213/24.88	/	213/22.07	/	213/22.54	213/22.54	213/24.88	213/22.38
IspE	936/30.88	936/30.88	936/30.77	936/30.45	936/30.24	936/30.24	/	936/30.84	936/30.31
IspF	486/34.16	486/34.16	486/33.74	486/33.74	486/33.13	486/33.54	486/33.54	486/34.02	486/33.49
IspH	1224/37.17	1224/37.17	1224/37.17	1236/36.41	1236/36.41	1236/36.33	1236/36.33	1224/37.17	1236/36.37
Glycolysis (EM phase)									
Glk	912/36.18	912/36.18	912/35.96	912/35.53	912/35.64	912/35.42	912/35.64	912/36.11	912/35.56
GPI	1581/35.29	1581/35.29	1581/35.17	1581/35.80	1581/35.86	1581/35.93	1581/35.93	1581/35.25	1581/35.88
ENO	1296/34.49	1296/34.49	1296/34.57	1296/34.57	1296/34.88	1296/34.72	1296/34.65	1296/34.52	1296/34.71
FBA	1068/39.23	1068/39.23	1068/39.51	1068/37.83	1068/38.01	1068/37.92	1068/38.11	1068/39.32	1068/37.97
GADPH	1035/34.49	1035/34.49	1035/34.59	1020/34.41	1020/34.31	1020/34.31	1020/34.31	1035/34.52	1020/34.34
Gpml	1587/33.59	1587/33.59	1587/33.52	1590/32.26	1590/32.58	1590/32.26	1590/32.14	1587/33.57	1590/32.31
PGK	1203/32.25	/	1203/32.50	1203/32.67	1203/32.17	1203/32.67	1203/32.59	1203/32.38	1203/32.53
PK	1752/34.93	1752/34.93	1752/35.10	1752/34.25	1752/34.30	1752/34.47	1752/34.47	1752/34.99	1752/34.37
TPI	723/34.02	723/34.02	723/34.16	717/33.89	717/33.47	717/33.61	717/33.75	723/34.07	717/33.68
Heme biosynthesis									
HemA	1293/35.03	1293/35.03	1293/35.03	1293/34.11	1293/34.34	1293/34.03	1293/34.11	1293/35.03	1293/34.15
HemB	957/34.80	957/34.80	957/34.59	954/36.16	954/35.85	954/36.06	954/36.06	957/34.73	954/36.03
HemD	744/36.69	744/36.69	744/36.42	765/32.68	765/32.68	765/32.81	765/32.81	744/36.6	765/32.75
HemE	1065/34.65	1065/34.65	1065/34.74	1065/35.40	1065/35.40	1065/35.21	1065/35.40	1065/34.68	1065/35.35
HemL	1302/36.71	1302/36.71	1302/36.71	1317/37.74	1317/37.59	1317/37.74	1317/37.66	1302/36.71	1317/37.68
HemN	1392/30.03	1392/30.03	1392/29.96	1404/29.63	1404/29.77	1404/29.70	1404/29.77	1392/30.01	1404/29.72
Pentose phosphate cycle									
G6PD	1530/37.25	1530/37.25	1530/37.39	1530/37.52	1530/37.45	1530/37.52	1530/37.52	1530/37.3	1530/37.5
PGD	1422/37.90	1422/37.90	1422/37.83	1422/38.40	1422/38.40	1422/38.12	1383/37.96	1422/37.88	1412.25/38.22
RPE	687/35.23	687/35.23	687/35.95	687/35.66	687/35.66	687/35.81	687/35.81	687/35.47	687/35.74
RpiA	702/36.04	702/36.04	702/35.75	702/35.33	702/35.75	702/35.61	702/35.47	702/35.94	702/35.54
TalA	981/33.54	981/33.54	981/33.54	981/35.58	981/35.27	981/35.47	981/35.58	981/33.54	981/35.48
TktA	2013/39.00	2013/39.00	2013/38.85	2013/38.9	2013/38.55	2013/38.75	2013/38.6	2013/38.95	2013/38.7
Proline biosynthesis									
ProB	1131/32.63	1131/32.63	/	1128/31.56	1128/31.29	1128/31.47	1128/31.47	1131/32.63	1128/31.45
Pyrimidine biosynthesis									
CarA	1152/34.29	1152/34.29	1152/34.38	1146/34.21	1146/34.64	1146/34.47	/	1152/34.32	1146/34.44
CarB	3246/35.40	3246/35.40	3246/35.37	2115/33.95	3246/34.81	3246/34.87	3246/34.87	3246/35.39	2963.25/34.63
PyrF	699/35.48	699/35.48	699/34.62	699/34.76	699/35.05	699/34.91	699/35.19	699/35.19	699/34.98
PyrI	984/34.35	984/34.35	984/34.04	987/35.26	987/35.16	987/35.26	987/35.26	984/34.25	987/35.24
PyrC	1269/34.59	1269/34.52	1269/34.52	1269/34.36	1269/34.44	1269/34.28	1269/34.44	1269/34.54	1269/34.38
PyrD	1140/31.05	1140/31.05	1140/31.14	1152/31.6	1140/31.58	1152/31.6	1152/31.51	1140/31.08	1149/31.57
Serine biosynthesis									
SerA	1578/36.44	1578/36.44	1578/36.19	1578/36.63	1578/36.38	1578/36.63	1578/36.57	1578/36.36	1578/36.55
Tetrahydrofolate biosynthesis									
FolB	369/30.08	369/30.08	369/30.35	360/29.17	360/28.89	360/29.44	360/29.44	369/30.17	360/29.24
FolC	843/33.69	843/33.69	843/33.69	843/33.69	843/33.57	843/33.57	843/33.57	843/33.69	843/33.6
FolE	462/35.93	708/34.75	708/34.6	711/35.44	711/35.44	711/35.58	711/35.44	626/35.09	711/35.48
Threonine biosynthesis									
Asd	1014/34.02	1014/34.02	1014/33.63	1014/35.60	1014/35.90	1014/35.60	1014/35.90	1014/33.89	1014/35.75
Hom	1290/33.64	1290/33.64	1290/33.88	1299/32.87	1299/32.87	1299/32.87	1299/32.72	1290/33.72	1299/32.83
ThrB	918/33.77	918/33.77	918/33.66	972/33.85	972/33.95	972/33.85	972/33.64	918/33.73	972/33.82

*Note:* / denotes the gene with incomplete sequence, which might be missing or pseudo.

#### 3.3.1 Informative SNPs and INDELs of the Metabolic‐Pathway Genes Among the Seven UCYN‐A Strains

The average GC content of the 40 genes across the seven strains fell into 23.38% (Dxr) – 38.81% (TktA), with an overall mean and standard deviation of 34.40% ± 2.81%. Specifically, at the intergroup level, the GC content of all the 40 genes was highly similar between the UCYN‐A1 and UCYN‐A2 strains, with differences ranging from 0.09% (ThrB and FolC) – 3.85% (HemD) (Table 5).

**FIGURE 2:**
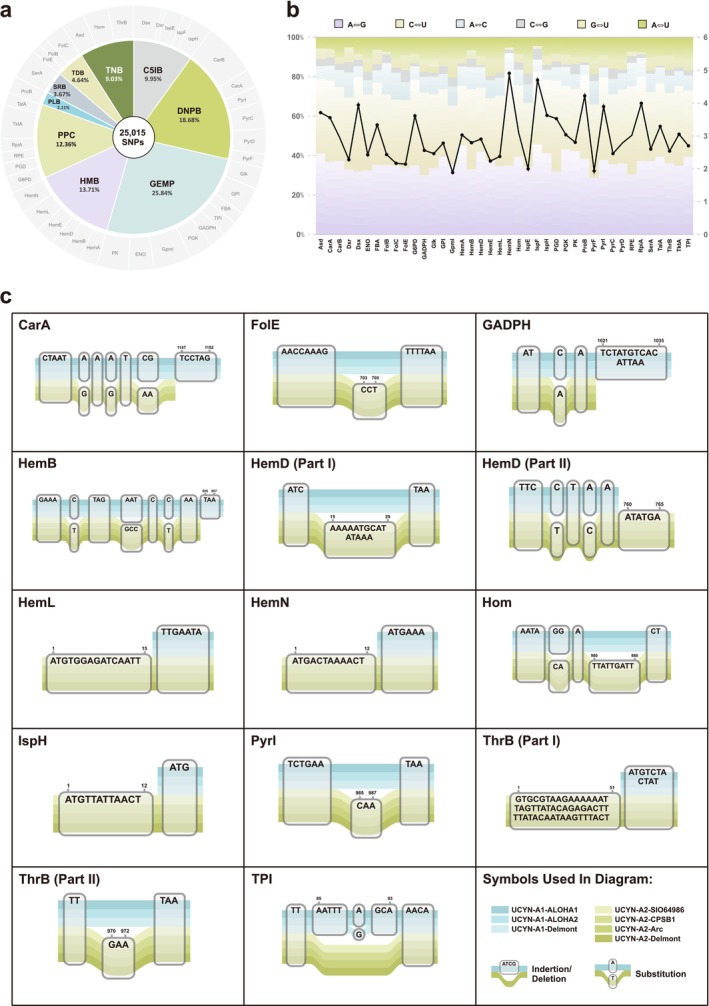
(a) Proportions of the SNPs from the nine pathway genes. (b) Substitution composition of the 40 metabolic‐pathway genes. The bar chart represented the percentage of different substitution types, while the dot‐line plot showed the ratio of transitions to transversions. The left y‐axis was for the bar chart, and the right y‐axis was for the dot‐line plot. (c) Specific illustrations for the 14 strain‐specific INDELs.

Comparative SNP analyses allowed the depiction of the SNP map for each of the nine pathways. With UCYN‐A1‐ALOHA1 as reference, a total of 25,015 SNPs were identified from the four UCYN‐A2 strains. Comparative analyses were implemented at three levels. First, at the gene level, among the 40 genes, CarB accounted for the most SNPs (1622, 6.48%), followed by Gpml (1140, 4.56%) and TktA (1010, 4.04%). In contrast, Dxr (118, 0.47%), FolE (264, 1.06%), and IspF (274, 1.10%) had the fewest SNPs (Table S5 and Figure 2a). Second, at the pathway level, the Serine Biosynthesis pathway featured the highest average number of SNPs per constituent gene (918), followed by the De Novo Pyrimidine Biosynthesis pathway (779) and the Glycolysis (EM phase) pathway (718) (Table S5). Third, at the strain level, negligible differences were observed among the four UCYN‐A2 strains, with SNP counts ranging from 6020 (UCYN‐A2‐Delmont) to 6382 (UCYN‐A2‐Arc). More specifically, the ratio of transition to transversion (ts/tv) for each of the 40 genes was over 1, with a range of 1.89 (Gmpl) to 4.87 (HemN) (Figure 2b). This result demonstrated that the transitions occupied the great majority of the SNPs.

In addition to single‐site mutations, we also explored the INDELs within the 40 genes between the UCYN‐A1 and UCYN‐A2 groups. As clearly presented in Figure 2c, a total of 14 informative intergroup INDELs were identified from 12 genes. These INDELs ranged in size from 3 (FolE, HemB, PyrI, ThrB, and TPI) to 51 bp (ThrB). Furthermore, with UCYN‐A1 as reference, most of these INDELs were insertions shared by all four UCYN‐A2 strains (74.58%). Deletions for the four genomes were only found in CarA, GADPH, HemB, and TPI.

#### 3.3.2 Diverse Codon Usage Patterns Between UCYN‐A1 and UCYN‐A2 Groups

The first focus of our CUB analyses was the RSCU calculations. For all the 40 genes, collectively, the consensus sequences of UCYN‐A1 possessed 1032 codons with RSCU values exceeding 1, while UCYN‐A2 strains showed a slightly lower count of 1026 such codons (Table S6). To compare RSCU patterns between the two groups, we defined codons that were preferred (RSCU > 1) in one group but unpreferred (RSCU ≤ 1) in the other as significantly variable codons (SVCs). Among the analyzed genes, FolB contained the highest number of SVCs (12), followed by Dxr (11), TPI (10), and RpiA (10). In contrast, CarB was the only gene with no SVCs (Table S6 and Figure S1). Interestingly, our results revealed strong intergroup differences in the terminal codon choices among 15 of the total 40 examined genes (Table S6). Specifically, for these 15 genes, the favorite terminal codon in the UCYN‐A1 group is TAG (7), followed by TAA (6) and TGA (2). However, for UCYN‐A2, the preference order is TAA (8), TGA (4), and TAG (3).

**FIGURE 3:**
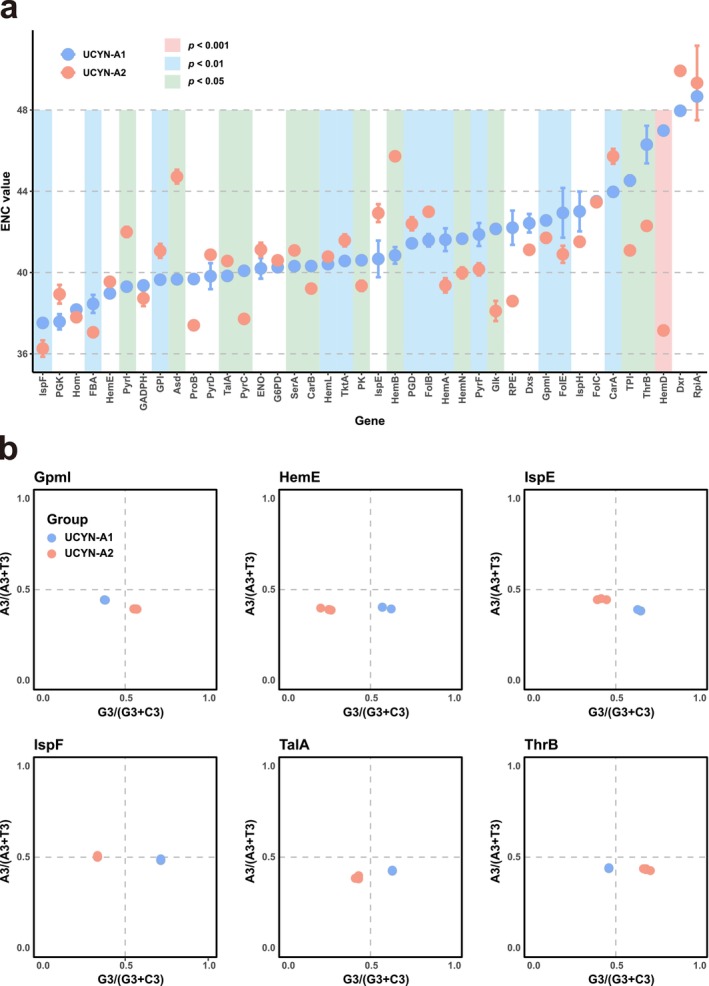
(a) ENC statistics for the metabolic‐pathway genes of the UCYN‐A strains, with the UCYN‐A1 and UCYN‐A2 marked with different colors. (b) PR2 plots of six genes that could clearly distinguish the A1 and A2 strains.

ENC index was further employed to measure absolute synonymous codon bias. In the UCYN‐A1 group, the three genes with the lowest ENC values were IspF (37.50 ± 0.00), PGK (37.58 ± 0.37), and Hom (38.18 ± 0.13), while those with the highest values were HemD (46.99 ± 0.01), Dxr (47.96 ± 0.00), and RpiA (48.66 ± 0.17). Although the UCYN‐A2 shared the lowest‐ and highest‐ENC genes with UCYN‐A1, a different pattern of the second and third lowest and highest were different, traced in FBA (37.06 ± 0.23) and HemD (37.15 ± 0.09), as well as RpiA (49.33 ± 1.83) and Dxr (49.93 ± 0.02), respectively (Table 6). Significantly, we conducted specific intergroup comparisons for each gene. *F*‐tests were primarily performed to assess whether the ENC variances of the two groups were equal. Based on the results, *t*‐tests were subsequently conducted. As clearly shown in Table 6 and Figure 3a, over half (25) of the 40 genes harbored significant differences (*p* < 0.05) for the ENC values between the UCYN‐A1 and UCYN‐A2 groups. Most notably, an extremely significant difference was detected in the ENC of HemD (*p* < 0.001), with a difference in average values of 9.84.

**TABLE 6:** ENC values for the 40 key‐metabolic‐pathway genes in the UCYN‐A1 and UCYN‐A2 groups.

Gene	ENC values (mean ± SD)		*p* value	Gene	ENC values (mean ± SD)		*p* value
	UCYN‐A1	UCYN‐A2			UCYN‐A1	UCYN‐A2	
Asd	39.67 ± 0.10	44.72 ± 0.34	< 0.01	HemN	41.66 ± 0.15	39.98 ± 0.27	< 0.01
CarA	43.97 ± 0.12	45.72 ± 0.37	< 0.05	Hom	38.18 ± 0.13	37.80 ± 0.20	> 0.05
CarB	40.32 ± 0.16	39.21 ± 0.13	< 0.01	IspE	40.67 ± 0.90	42.92 ± 0.44	> 0.05
Dxr	47.96 ± 0.00	49.93 ± 0.02	> 0.05	IspF	37.52 ± 0.00	36.26 ± 0.40	< 0.05
Dsx	42.43 ± 0.46	41.12 ± 0.05	> 0.05	IspH	43.01 ± 0.98	41.52 ± 0.24	> 0.05
ENO	40.20 ± 0.51	41.13 ± 0.34	> 0.05	PGD	41.44 ± 0.11	42.40 ± 0.33	< 0.05
FBA	38.46 ± 0.44	37.06 ± 0.23	< 0.05	PGK	37.58 ± 0.37	38.93 ± 0.46	> 0.05
FolB	41.59 ± 0.31	42.99 ± 0.21	< 0.05	PK	40.60 ± 0.21	39.35 ± 0.25	< 0.01
FolC	43.50 ± 0.24	43.46 ± 0.22	> 0.05	ProB	39.68 ± 0.00	37.40 ± 0.10	> 0.05
FolE	42.94 ± 1.22	40.90 ± 0.42	< 0.05	PyrC	40.09 ± 0.19	37.72 ± 0.09	< 0.01
G6PD	40.26 ± 0.16	40.60 ± 0.11	> 0.05	PyrD	39.82 ± 0.64	40.88 ± 0.21	> 0.05
GADPH	39.37 ± 0.03	38.73 ± 0.38	> 0.05	PyrF	41.87 ± 0.57	40.16 ± 0.30	< 0.05
Glk	42.15 ± 0.03	38.11 ± 0.50	< 0.01	PyrI	39.30 ± 0.03	42.00 ± 0.25	< 0.01
GPI	39.65 ± 0.08	41.07 ± 0.34	< 0.05	RPE	42.21 ± 0.84	38.59 ± 0.11	> 0.05
Gpml	42.56 ± 0.08	41.71 ± 0.23	< 0.05	RpiA	48.66 ± 0.17	49.33 ± 1.83	> 0.05
HemA	41.62 ± 0.56	39.37 ± 0.36	< 0.05	SerA	40.31 ± 0.10	41.09 ± 0.13	< 0.01
HemB	40.84 ± 0.41	45.72 ± 0.21	< 0.01	TalA	39.83 ± 0.05	40.57 ± 0.20	< 0.01
HemD	46.99 ± 0.01	37.15 ± 0.09	< 0.01	ThrB	46.30 ± 0.92	42.30 ± 0.21	< 0.01
HemE	38.97 ± 0.16	39.55 ± 0.14	> 0.05	TktA	40.57 ± 0.05	41.58 ± 0.30	< 0.05
HemL	40.41 ± 0.01	40.78 ± 0.12	< 0.05	TPI	44.53 ± 0.25	41.09 ± 0.14	< 0.01

We additionally implemented PR2 analysis to gain insights into the CUB of the four‐fold degenerate codons (Table S7). Based on the calculated third codon position AT and GC biases (Table 7), we observed rather wide ranges for the mean values, from 0.34 (PGD and Dxr) to 0.51 (HemD) for AT bias, and from 0.10 (Dxr) to 0.59 (ThrB) for GC biases. Note that the different quadrant locations of the plotted dots indicated different biases, with Quadrant I, II, III, and IV representing AG‐, AC‐, TC‐, and TG‐biases, respectively. At the intergroup level, it is worth noting that for most of the 40 genes (85%), the UCYN‐A1 and UCYN‐A2 strains tended to have similar patterns for AT/GC bias. However, importantly, we still detected distinct disparities in six genes between the two groups (Figure 3b). Interestingly, all the disparities were confined to GC bias. The results can be more specifically categorized into two types: (1) the UCYN‐A1 group harbored obviously stronger C‐bias than UCYN‐A2 in the Gpml and ThrB genes, and (2) the opposite scenario was found in HemE, IspE, IspF, and TalA.

**TABLE 7:** AT and GC biases for the 40 key‐metabolic‐pathway genes in the UCYN‐A strains.

Gene	A3/(A3 + T3) (mean ± SD)	G3/(G3 + C3) (mean ± SD)	Gene	A3/(A3 + T3) (mean ± SD)	G3/(G3 + C3) (mean ± SD)
Asd	0.42 ± 0.02	0.32 ± 0.02	HemN	0.39 ± 0.01	0.36 ± 0.03
CarA	0.48 ± 0.01	0.35 ± 0.09	Hom	0.42 ± 0.00	0.38 ± 0.07
CarB	0.41 ± 0.02	0.32 ± 0.02	IspE	0.42 ± 0.03	0.53 ± 0.13
Dxr	0.34 ± 0.01	0.10 ± 0.14	IspF	0.49 ± 0.01	0.50 ± 0.20
Dsx	0.38 ± 0.00	0.31 ± 0.01	IspH	0.40 ± 0.02	0.35 ± 0.03
ENO	0.37 ± 0.01	0.36 ± 0.04	PGD	0.34 ± 0.00	0.33 ± 0.07
FBA	0.35 ± 0.02	0.39 ± 0.05	PGK	0.35 ± 0.01	0.34 ± 0.12
FolB	0.38 ± 0.01	0.12 ± 0.16	PK	0.46 ± 0.02	0.38 ± 0.04
FolC	0.46 ± 0.01	0.27 ± 0.05	ProB	0.49 ± 0.02	0.37 ± 0.05
FolE	0.40 ± 0.04	0.32 ± 0.05	PyrC	0.46 ± 0.00	0.38 ± 0.02
G6PD	0.40 ± 0.01	0.37 ± 0.02	PyrD	0.42 ± 0.00	0.47 ± 0.03
GADPH	0.42 ± 0.02	0.30 ± 0.02	PyrF	0.44 ± 0.01	0.54 ± 0.19
Glk	0.45 ± 0.01	0.40 ± 0.10	PyrI	0.43 ± 0.02	0.46 ± 0.09
GPI	0.43 ± 0.01	0.49 ± 0.02	RPE	0.42 ± 0.01	0.19 ± 0.05
Gpml	0.41 ± 0.03	0.48 ± 0.10	RpiA	0.40 ± 0.02	0.38 ± 0.13
HemA	0.47 ± 0.02	0.37 ± 0.03	SerA	0.41 ± 0.03	0.36 ± 0.04
HemB	0.46 ± 0.03	0.36 ± 0.06	TalA	0.40 ± 0.02	0.51 ± 0.11
HemD	0.51 ± 0.01	0.36 ± 0.11	ThrB	0.44 ± 0.01	0.59 ± 0.12
HemE	0.40 ± 0.01	0.39 ± 0.19	TktA	0.37 ± 0.01	0.36 ± 0.09
HemL	0.50 ± 0.01	0.35 ± 0.10	TPI	0.49 ± 0.01	0.37 ± 0.02

#### 3.3.3 Codon Aversion Motifs as Distinctive Markers for UCYN‐A Strain Identification

The codon aversion analysis revealed an important finding: No genes displayed the exactly same CAM patterns across these UCYN‐A strains (Table S8). More significantly, striking variations were observed in the CAMs of all the 40 genes, both at the intergroup and interstrain levels.

Of these genes, a total of 19 genes possessed the CAMs that could distinctly and completely differentiate the UCYN‐A1 from UCYN‐A2 groups (Table S8). Besides, the ecotype‐specific CAMs were determined in several genes, with CarA, CarB, Glk, HemE, IspH, ProB, RpiA, and TPI for the UCYN‐A1 group, as well as Asd, FolB, PyrI, and PyrF for the UCYN‐A2 group (Table S8).

We also discovered abundant strain‐specific CAMs scattering in various genes. Firstly, for the UCYN‐A1 group, the CAMs of FolE served as a perfect marker, which could distinguish all the three investigated strains (Table S8). For the UCYN‐A2 strains, only the CAMs with “less precise” discriminability were detected in several genes. For example, the codon aversion motifs in Glk could only distinguish UCYN‐A2‐CPSB1 and UCYN‐A2‐Delmont, as UCYN‐A2‐Arc and UCYN‐A2‐SIO64986 shared the same motif. In general, such “less precise” CAMs were observed in a total of 29 genes.

**FIGURE 4:**
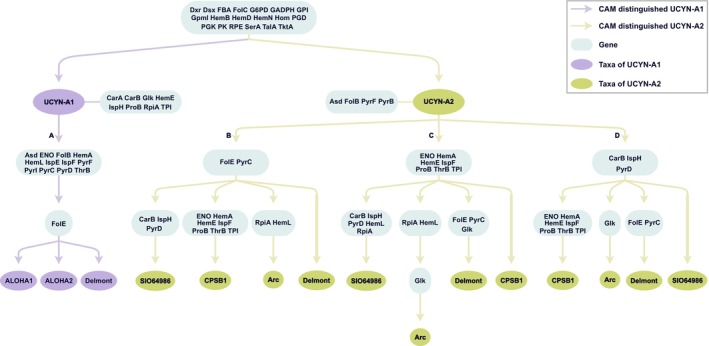
A comprehensive strain‐identification pathway for the seven UCYN‐A strains, established by the codon aversion motifs of the 40 genes. The light blue rounded rectangle highlighted the genes with distinctive CAMs in each identification step, with the specific CAMs documented in Table S8. A, B, C, and D denoted different pathways to identify specific strains.

Based on these findings, an exciting hypothesis emerged: the CAM discrimination for the two ecotypes, and even for each of the seven UCYN‐A strains, is logically achievable by our current findings. Here, we successfully established a comprehensive strain‐identification pathway for the involved seven UCYN‐A strains using the codon aversion motifs in the 40 genes. As clearly depicted in Figure 4, after distinguishing the two ecotypes by 19 listed genes, one strategy was developed to discriminate all the UCYN‐A1 strains (pathway A), while three strategies were established to differentiate UCYN‐A2 strains (pathway B, C, and D) strains. Note that this conclusion was still a progress report merely, there will remain uncertainty along with the increasing data in the future.

## 4 Discussion

### 4.1 Remarkable Genomic Divergences Between UCYN‐A1 and UCYN‐A2 Indicate Potential Species‐Level Differentiation

The circumscription of bacterial species has long been a topical issue. One effective method for this is evaluating the genome relatedness, for which the whole‐genome average nucleotide identity (ANI) has been widely recommended as a crucial tool (Jain et al. 2018). ANI can closely mirror DNA–DNA hybridization (DDH), the traditional standard in circumscribing bacterial taxa (Narsing Rao and Thamchaipenet 2024; Goris et al. 2007). Here we present the genome‐level ANI analysis between the seven examined UCYN‐A1 and UCYN‐A2 ecotypes. Strikingly, our results clearly show that all interecotype ANI values fell below 85%. Note that the work of Kantor et al. also displays similar ANI‐gap findings between UCYN‐A1 and UCYN‐A2 (Kantor et al. 2024). With typical ANI cutoffs at 85% and 95%, intergenome relatedness can be assigned into three levels: (1) obvious disparity (typically classified into two different species), (2) intermediate relatedness (less frequent, usually recognized as distinct species), and (3) strong relatedness (considered as the same species) (Konstantinidis and Tiedje 2005; Goris et al. 2007; Rodriguez‐R et al. 2021; Viver et al. 2024). It is important to note that the ecotype relationships of UCYN‐A1 and UCYN‐A2 have never been challenged before. However, based on our results, we highly recommend reevaluating the species allocation of UCYN‐A1 and UCYN‐A2. The following points, inferred from previous research, potentially support our conclusion.

The first point is the evolutionary fate of bacterial ecotypes. As background, it is the ecological cohesion (especially the periodic selection) that restricts genetic variability within certain ecotypes (Meglitsch 1954; Cohan 2001). Once the accumulated divergence of ecotypes escapes each other's cohesion, they will be unconstrained with respective evolutionary directions. Ultimately, such ecotypes will irreversibly diverge into separate species (Cohan 2002; De Queiroz 1998; Wiley 1978; Cohan 2001).

Secondly, among the multiple ecotypes of UCYN‐A, the UCYN‐A1 and UCYN‐A2 represent the most abundant strains (Turk‐Kubo et al. 2017). According to Cohan (Cohan 2001), speciation occurs more frequently in bacteria than in higher sexual eukaryotes. Such phenomena are mainly attributed to two factors: (1) the more “relaxed” requirements for bacterial speciation, which involve only ecological divergence (Cohan 1994), and (2) the typically vast population of bacteria, which allows for much greater genetic exchange than in macroorganisms (Cohan 2001).

Third, several case studies have utilized ANI threshold as a criterion for species circumscription. For instance, Nelkner et al. (2019) used a 95% ANI threshold to eliminate four strains from the species *Pseudomonas brassicacearum* . Similarly, an ANI threshold of 85% was applied to distinguish species‐level clades within the *Candidatus* Bgiplasma genus (Zhu et al. 2022).

Some limitations should also be clarified here. Although the ANI has been a widespread tool to discriminate bacteria species, the context is more complicated for the symbiotic UCYN‐As, which has undergone a quite large genome reduction with a large‐scale loss of some important genes (Tripp et al. 2010). This might impact the applicability of ANI in species discrimination for the seven UCYN‐A strains. Given the limited knowledge and data availability for their host nuclear genomes, we still cannot firmly hypothesize that the UCYN‐A1 and UCYN‐A2 have evolved into distinct species beyond ecotypes. However, regardless of these uncertainties, the species‐level divergent ANI values and the identified genomic disparities between the investigated UCYN‐A1 and UCYN‐A2 collectively highlight their substantial underlying ecological or functional differences.

### 4.2 Conserved INDEL Mutations Shed Light on the Relationships Between the UCYN‐A1 and UCYN‐A2 Groups

INDELs play a significant role in driving genome evolution (Williams and Wernegreen 2013), which can lead to shifts or disruptions in the reading frame and promote adaptive evolution (Leushkin et al. 2012; Tian et al. 2008; Vakhrusheva et al. 2011). In the case of endosymbiotic bacteria, INDEL patterns are usually influenced by the combined forces of a lost DNA repair system and relaxed purifying selection (Moran et al. 2008). Further, Williams and Wernegreen (2013) noted that purifying selection is the main constraint for coding‐region INDELs, by purging major INDELs and nonsynonymous mutations (Williams and Wernegreen 2012). Nevertheless, some INDELs can persist in PCG regions, particularly those with lengths that are multiples of three, which preserve the reading frame. In this study, we also discovered a consistent pattern between UCYN‐A1 and UCYN‐A2. Among the 2106 INDELs detected in the SCOGs, 84.95% (1790) met the multiple‐of‐three size condition. Significantly, it has been documented that such retaining frameshift‐inducing INDELs may avoid deleterious influence and thus escape purging pressure (Williams and Wernegreen 2013). These INDELs can potentially drive the interstrain protein divergence. Accordingly, the abundant INDELs identified here indicate a quite high level of protein variation between UCYN‐A1 and UCYN‐A2.

Aside from inducing protein divergence, INDELs have also been proposed as a strong tool for analyzing bacterial phylogeny. Utilizing 12 signature sequences, Gupta (2001) successfully determined the phylogenetic positions of 41 species representing major bacterial lineages. In our analysis, we identified plentiful lineage‐conserved INDELs within the SCOGs, with UCYN‐A2 strains sharing extremely high identity for small deletions, small insertions, and large insertions. This INDEL identity also largely occurred in the intergroup alignments of metabolic‐pathway genes. These observations significantly vindicate the species‐level divergence between UCYN‐A1 and UCYN‐A2. More importantly, the identified INDELs could be considered effective molecular markers for distinguishing between UCYN‐A1 and UCYN‐A2 at the intergroup level.

### 4.3 Indications From CUB Analyses Promote Our Understanding of the Evolution of UCYN‐A1 and UCYN‐A2

The RSCU index has long been considered a significant indicator for synonymous codon usage (Bi et al. 2023). The certain codons with RSCU > 1 are presumed to undergo positive biases and vice versa. For the UCYN‐A strains investigated here, we observed a dominant preference for codons ending with A or U. Notably, this A/U‐ending trend has been frequently reported in plants (Ding, Han, et al. 2022; Han, Bi, et al. 2022; Bi et al. 2023; Yang et al. 2023; Zhang, Wang, et al. 2024) and bacteria (Liu et al. 2016; Chen et al. 2018). However, some exceptions have been observed, such as in the case of the radioresistant bacterial genome, which favors codons ending with G/C over A/U (Dilucca et al. 2020). As documented previously, the mutational pressure usually drives bacterial genomes toward higher AT content (Hershberg and Petrov 2010; Hildebrand et al. 2010). Moreover, this AT‐biased genome is generally thought to be the major reason for the A/U‐ending trend (Liu et al. 2016; Bi et al. 2023).

In the present study, we proposed a novel method for assessing interlineage RSCU divergence—significantly variable codons (SVCs). Based on this approach, we identified 1–12 SVCs across all 40 investigated metabolic‐pathway genes between UCYN‐A1 and UCYN‐A2. This result indicated a certain intergroup disparity in codon usage. It is noteworthy that patterns of synonymous codons can serve as molecular signatures of bacterial evolutionary adaptation to environmental shifts (Liu et al. 2016). The determined SVCs reported here can be highly informative for understanding the evolutionary divergence between UCYN‐A1 and UCYN‐A2 strains.

Another important finding from the RSCU analysis is that UCYN‐A1 and UCYN‐A2 display different termination‐codon choices in 15 genes. Bacteria commonly have three termination codons: UAA, UAG, and UGA, decoded by different release factors (RF1 for the former two, and RF2 for UAA and UGA) (Scolnick et al. 1968; Milman et al. 1969). Importantly, the choice of distinct terminal codon may reflect differences in translational termination machinery. An interesting finding was made by Wei et al. (2016), who noted that the genes with different expression levels exhibit certain biases in stop‐codon choices across 14 bacteria species, including Cyanobacteria. Most significantly, UAA appears to be universally favored in genes with high expression, while UGA is preferred in genes with low expression. Accordingly, our findings, which reveal intergroup differences in termination‐codon choices for 15 genes, may suggest divergent expression levels of these metabolic pathway proteins.

The ENC parameter is an efficient tool for accurately assessing the degree of CUB in a given gene (Parvathy et al. 2022). Furthermore, there is a presumed negative relationship between the ENC value and the extent of CUB or gene expression level (Kandeel et al. 2020; Wang et al. 2018; NaIR et al. 2014). Based on our results, the metabolic‐pathway genes from both UCYN‐A1 and UCYN‐A2 similarly harbored an overall weak codon preference. Nevertheless, significant interlineage ENC disparities were observed across the 25 genes, especially in HemD. Such comparative divergence might indicate a certain degree of genetic variability among these UCYN‐A strains (Barbhuiya et al. 2020). Note that similarly weak codon preference is also detected in *Actinobacteria* genomes (Lal et al. 2016), *Bletilla* (Orchidaceae) cpDNA (Han, Wang, et al. 2022), HSP60 genes of birds (Yang et al. 2021), and others. Most importantly, the benefits of this low CUB can be attributed to facilitating efficient replication and allowing organisms to employ variable codons for translation (Jenkins and Holmes 2003). Furthermore, the overall high ENC values demonstrated rather weak selective forces in the involved seven UCYN‐A strains. Note that it has been hypothesized that in small populations or symbionts, the decrease of selection and weakness of codon usage bias usually comes with the more dominant impact of genetic drift (Duret 2002; Muto and Osawas 1987; Plotkin and Kudla 2011). Moran et al. (2008) also illustrated increased genetic drift in symbionts, which might be due to relaxed selection and population–structure changes.

The PR2 bias is an excellent indicator for estimating the forces of mutation and natural selection in forming the codon preference pattern. Theoretically, the dot that is centered on the plot demonstrates a mutation–selection balance power (Parvathy et al. 2022). For the examined UCYN‐A strains, we observed a dominative preference of pyrimidine over purine, with only a few exceptions. Similar phenomena have been reported in parasitic protozoa (six *Eimeria* genomes, Apicomplexa) (Zhao and Zhang 2024) and plant plastids (12 *Solanum* plastomes, Solanaceae) (Zhang et al. 2018). Meanwhile, the opposite case was also detected in several chloroplast genes of Saxifragales (Bi et al. 2023). Regardless of the direction of bias, these base tendencies evidently denote that natural selection and mutation/drift pressure remarkably impact the CUB pattern of the organisms.

### 4.4 Striking Resolution of CAM for Distinguishing All Examined Strains of UCYN‐A1 and UCYN‐A2

In addition to the most widely studied codon usage, codon aversion has recently attracted increasing interest in molecular evolution research. The concept of codon aversion was first established by Miller et al. (2017). They afterwards vindicated the highly phylogenetic‐conserved characteristic of CAM using 12,337 taxa covering all life domains (Miller et al. 2020). We have previously made a lot of efforts to explore the ability of CAM in disentangling the evolutionary affinities of various lineages involving plastid genes of plants, such as *Bletilla* (Han, Wang, et al. 2022) (Orchidaceae), and *Aeonium*, *Monanthes* (Han, Bi, et al. 2022), and *Crassula* (Ding, Han, et al. 2022) (Crassulaceae), as well as Aves mitochondrial genes (Sturnidae *sensu lato*) (Han, Ding, et al. 2024). All the above studies collectively demonstrate that CAM can be regarded as special “signatures” for various taxa or even genes.

Here, we first focused on the bacterial codon aversion pattern. As anticipated, codon aversion still has quite high resolution in recognizing the involved seven UCYN‐A strains. The most innovative achievement is the establishment of a strain‐identification map based on strain‐specific averse codons. This result not only enlightens us to novel molecular markers at both intergroup and interstrain levels, but also credibly reinforces the ability of CAM in understanding the evolutionary relationships within bacteria.

## 5 Conclusion

This study conducted comprehensive comparisons among the seven strains from nitroplast‐evolved UCYN‐A2 and its closest relative, UCYN‐A1. The comparisons included overall gene content, nucleotide compositions of all SCOGs, sequence variations, as well as CUB and CAM patterns of the key metabolic‐pathway genes in nitroplast. Despite the similar genomic organizations, we revealed a considerable degree of disparity between these two lineages. The most evident species‐level divergence is that the ANI values between UCYN‐A1 and UCYN‐A2 obviously fell below 85%. Meanwhile, abundant lineage‐specific INDELs of SCOGs and metabolic pathways reinforced the interlineage genetic differences. Furthermore, we dedicated lots of effort to the comparative analyses of the 40 metabolic‐pathway genes. Numerous SNPs and 14 high‐informative INDELs shed light on the high heterogeneity between the two groups. Also, CUB analyses depicted the general picture of UCYN‐A evolutionary tendencies. RSCU indicated an AT bias at the third codon position, ENC enlightened a certain degree of interlineage genetic variability, and PR2 plots denoted the important role of selection pressure across evolution. Most strikingly, one important innovation of this work is concentrated on CAM analyses, which established a strain‐identification map for all seven strains. It is noteworthy that this is the first in‐depth case study for bacterial CAMs. Based on these various evidences, we strongly recommend reconsidering the species allocation of UCYN‐A1 and UCYN‐A2. Altogether, the above conclusions not only clearly provided a series of specific molecular markers for both UCYN‐A1 and UCYN‐A2, but also offer fresh insights into understanding the evolution of UCYN‐A.

## Author Contributions

**Shiyun Han:** data curation (equal), formal analysis (equal), methodology (equal), writing – original draft (equal). **Sijia Zhang:** investigation (equal). **Wen Ge:** resources (equal). **Jianke Yang:** software (equal). **Hui Peng:** investigation (equal). **Jinming Gao:** software (equal). **Mengsa Zhang:** formal analysis (equal). **Yingying Xiao:** validation (equal). **Dongsheng Du:** project administration (equal). **Xianzhao Kan:** conceptualization (equal), funding acquisition (equal), project administration (equal), supervision (equal), writing – review and editing (equal).

## Conflicts of Interest

The authors declare no conflicts of interest.

## Supporting information


**Figure S1.** Comparisons of RSCU values of the metabolic‐pathway genes between UCYN‐A1 and UCYN‐A2 strains. With each gene forming a group, the top and the bottom one represented UCYN‐A1 and UCYN‐A2, respectively.

**Table S1.** Data information for the seven published UCYN‐A genomes.

**Table S2.** Protein IDs of the 1207 orthologous genes identified among the seven UCYN‐A genomes.

**Table S3.** Protein IDs of the 1020 single‐copy orthologous genes identified among the seven UCYN‐A genomes.

**Table S4.** INDELs identified in the 1020 single‐copy orthologous genes among the seven UCYN‐A strains.

**Table S5.** SNPs identified in the 40 metabolic‐pathway genes from the four UCYN‐A2 strains, with UCYN‐A1 as reference.

**Table S6.** RSCU index of the 40 key‐metabolic‐pathway genes.

**Table S7.** PR2 analysis for the gene Asd.

**Table S8.** The identified codon aversion motifs in the 40 key‐metabolic‐pathway genes from each UCYN‐A strain.

## Data Availability

The original data that reported in this paper has been deposited in Figshare with the DOI of 10.6084/m9.figshare.28200554.v1 and 10.6084/m9.figshare.28200599.v1.
