# Supplementary figures and images for: Comparative Genomics Insights Into the Evolutionary Disparities Between Nitroplast‐Evolved Ecotype UCYN‐A2 and Its Closest Relative UCYN‐A1
Source: Ecol Evol. 2025 Jul 7;15(7):e71739. doi: 10.1002/ece3.71739 (PMC12234771; doi:10.1002/ece3.71739)

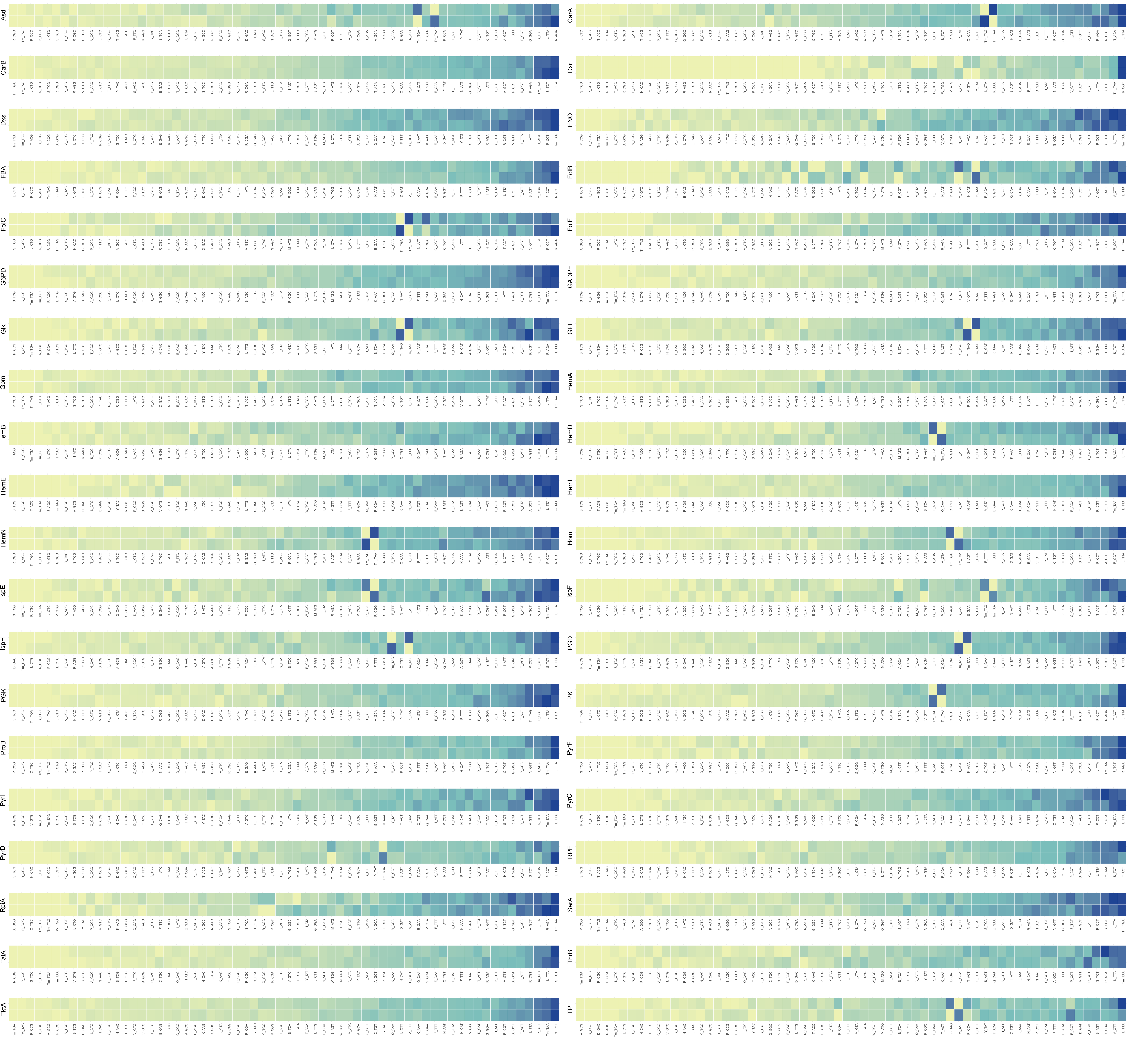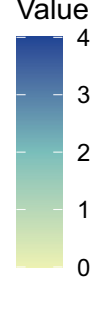

Supplement: Supplementary file 1 — Figure S1. Comparisons of RSCU values of the metabolic‐pathway genes between UCYN‐A1 and UCYN‐A2 strains. With each gene forming a group, the top and the bottom one represented UCYN‐A1 and UCYN‐A2, respectively. [file ECE3-15-e71739-s008.pdf]
